# Transcatheter Tricuspid Valve Replacement in a Patient With Small Right Atrium and Multiple Device Leads

**DOI:** 10.1016/j.jaccas.2026.107518

**Published:** 2026-03-25

**Authors:** Matthew Bauer, Jacob M. Mishell, Emily Perdoncin, Ivy Ku, Gordon Leung, Jonathan Zaroff, Anna Sannino, Andrew N. Rassi

**Affiliations:** a Kaiser Permanente-San Francisco Medical Center, San Francisco, California, USA; b Baylor Scott & White Research Institute, Dallas, Texas, USA

**Keywords:** cardiac pacemaker, imaging, right ventricle, tricuspid valve, ultrasound, valve repair, valve replacement

## Abstract

**Background:**

Severe tricuspid regurgitation (TR) in patients with small right atrial (RA) or right ventricular dimensions poses a significant challenge for transcatheter tricuspid valve replacement (TTVR).

**Case Summary:**

A 39-year-old woman with prior coronary artery bypass grafting, mitral valve replacement, and *LMNA* cardiomyopathy with a dual-chamber implantable cardioverter-defibrillator presented with torrential TR. She was deemed to be a poor surgical candidate, and TTVR with the EVOQUE system was aborted due to insufficient RA height. She underwent TTVR with the Laplace system. Imaging confirmed resolution of TR, and the patient had notable and sustained improvements in her functional capacity and quality of life at 6-month follow-up.

**Discussion:**

This case demonstrates the feasibility of the Laplace TTVR system for treating severe TR in a patient with small RA/right ventricular size that precluded the use of currently available commercial TTVR devices.

**Take-Home Message:**

Novel TTVR systems may provide additional options for treating TR in patients with challenging anatomy.

## Visual Summary

**Figure:**
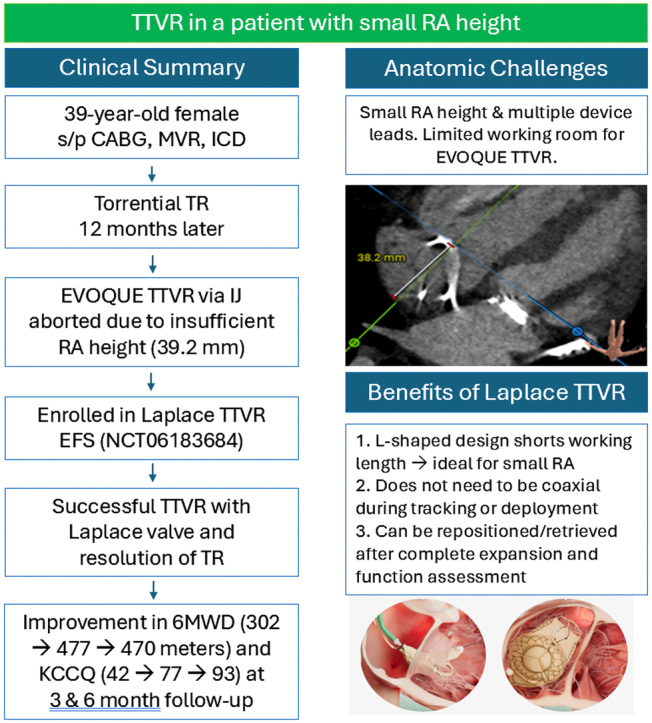

**Visual Summary:**
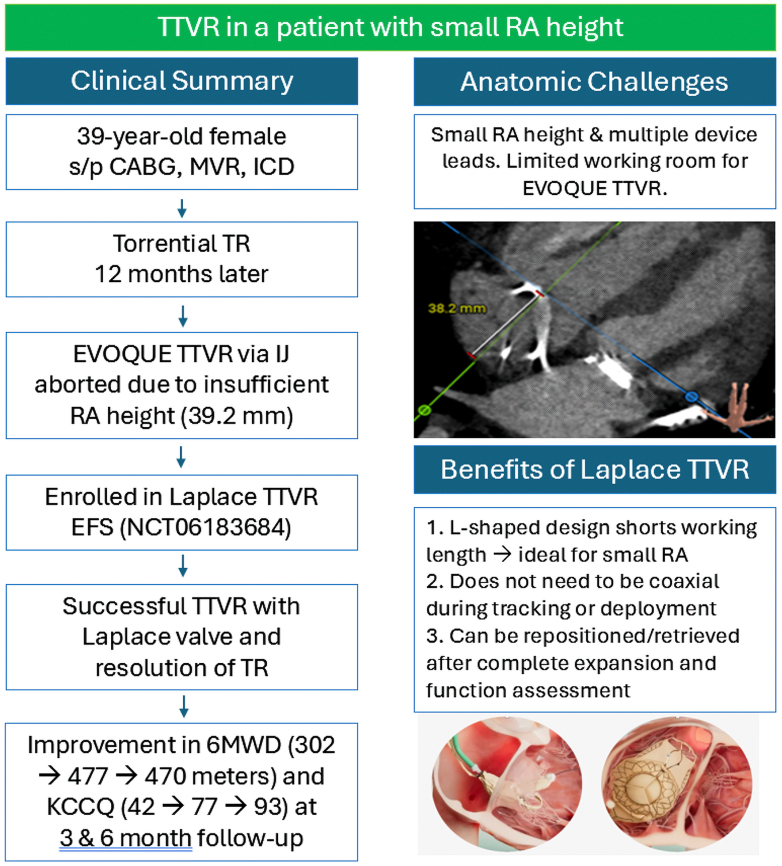
Summary of Clinical Course, Anatomic Challenges, and Potential Benefits of the Laplace TTVR 6MWD = 6-minute walk distance; CABG = coronary artery bypass grafting; EFS = early feasibility study; ICD = implantable cardioverter-defibrillator; IJ = internal jugular; KCCQ = Kansas City Cardiomyopathy Questionnaire; MVR = mitral valve replacement; RA = right atrial/right atrium; s/p = status post; TR = tricuspid regurgitation; TTVR = transcatheter tricuspid valve replacement

## History of Presentation

A 39-year-old woman presented for evaluation of torrential, symptomatic tricuspid regurgitation (TR) 12 months after coronary artery bypass grafting and mechanical mitral valve replacement.

**Take-Home Messages**

• Patients with severe TR and anatomical constraints that preclude the use of approved devices might be candidates for treatment with novel TTVR systems that address common anatomical barriers, with similar improvements in symptoms and quality of life.
• As with all investigational devices, long-term assessment of safety and durability is critical.

## Medical History

The patient's medical history was notable for a prior hospitalization for acute decompensated heart failure with severe functional mitral regurgitation and non–ST-segment elevation myocardial infarction, for which she underwent 3-vessel coronary artery bypass grafting, mechanical mitral valve replacement, and left atrial appendage exclusion. Her postoperative course was complicated by cardiogenic shock and digital ischemia secondary to prolonged vasopressor requirement. Genetic evaluation confirmed *LMNA* cardiomyopathy and she underwent dual-chamber implantable cardioverter-defibrillator (ICD) with atrial and ventricular leads for primary prevention of sudden cardiac death.

Over the next 12 months, she developed torrential symptomatic TR. Despite a revision of ICD leads and medical therapy, she continued to have symptoms of abdominal distension and lower extremity edema. She was evaluated for surgical repair vs transcatheter tricuspid valve repair or replacement.

## Differential Diagnosis

Possible etiologies for her TR included functional/secondary TR due to annular enlargement associated with her cardiomyopathy, primary TR from abnormal leaflet morphology or motion, and iatrogenic TR in the setting of a recently implanted ICD lead traversing the tricuspid valve. Of note, a prior attempt at lead revision was unsuccessful in relieving her TR.

## Investigations

**Figure 1:**
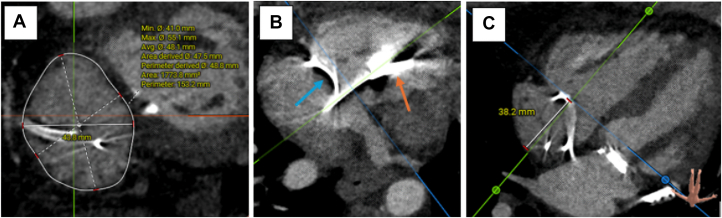
Screening Computed Tomography Assessment (A) Annulus sizing, (B) location of atrial lead (blue arrow) and ventricular lead (orange arrow), and (C) right atrial height measurement.

**Figure 2:**
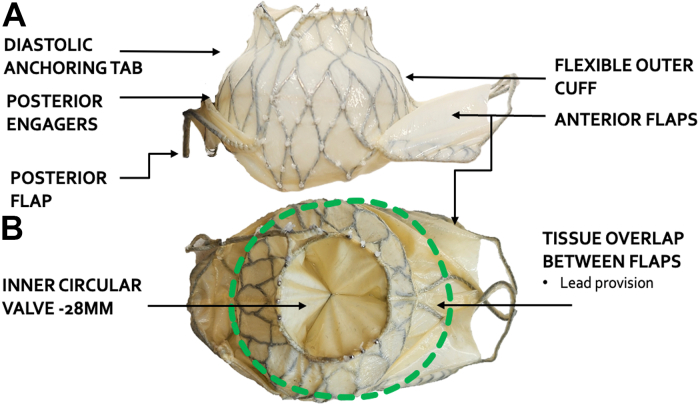
Laplace TTVR Laplace transcatheter tricuspid valve replacement valve in (A) lateral view and (B) en face view. Green line represents native annulus.

**Figure 3:**
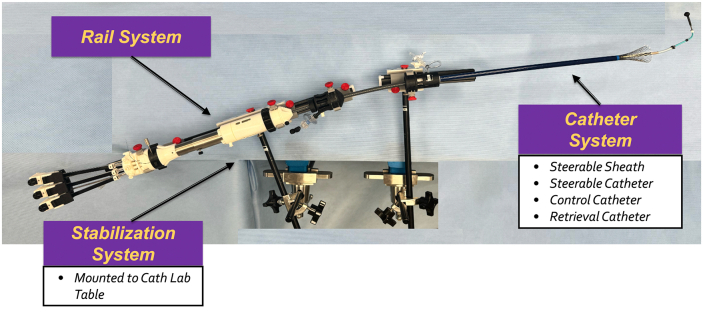
Laplace Transcatheter Tricuspid Valve Replacement Delivery System

Transthoracic echocardiography (TTE) demonstrated normal left ventricular and right ventricular (RV) size and function, with torrential TR (vena contracta: >21 mm). The tricuspid annular planar systolic excursion to pulmonary arterial systolic pressure ratio of 0.44 mm/mm Hg suggested appropriate RV to pulmonary arterial coupling. The RV fractional area change measured 40%, and the RV strain was −16.8%. Computed tomography demonstrated an enlarged tricuspid annulus (diastolic perimeter: 153 mm; maximum diameter: 55.1 mm) (Figure 1). The RV ICD lead was mobile and not adhered to the ventricular wall, with a course through the posterior/septal commissure. The RA lead formed a large loop near the tricuspid annulus. No tricuspid leaflet prolapse was observed. The patient was evaluated by a multidisciplinary heart team and deemed high risk for isolated surgical tricuspid repair due to her genetic cardiomyopathy and recent postoperative course. Transcatheter tricuspid edge-to-edge repair (T-TEER), transcatheter tricuspid valve replacement (TTVR), and caval valve implantation (CAVI) were all considered. Given the patient's young age, torrential TR, and normal RV function, the heart team desired complete resolution of her TR if technically feasible. Although T-TEER can reduce TR to moderate or less in many patients, complete resolution of torrential TR is unlikely, whereas CAVI is a primarily palliative treatment option reserved for patients without viable alternatives. Given this, she was initially considered for TTVR using the EVOQUE TTVR system (Edwards Lifesciences); however, there was insufficient right atrial (RA) height for a standard transfemoral approach. An attempt at TTVR using EVOQUE via a transjugular approach was attempted but aborted due to insufficient working room related to the small size of the patient's right atrium. After a period of recovery, she consented for participation in the Laplace TTVR Early Feasibility Study (Early Feasibility Study [EFS] Laplace Transcatheter Tricuspid Valve Replacement [TTVR] System; NCT06183684) with the intent that TTVR could successfully be performed using the Laplace TTVR system. The Laplace system is constructed from a Nitinol frame and cross-linked porcine pericardium. The TTVR system consists of a 28-mm inner valve that expands into an outer flexible frame (Figure 2), and the delivery system, which includes a rail system, stabilization system, and a catheter system (Figure 3).

## Management

**Figure 4:**
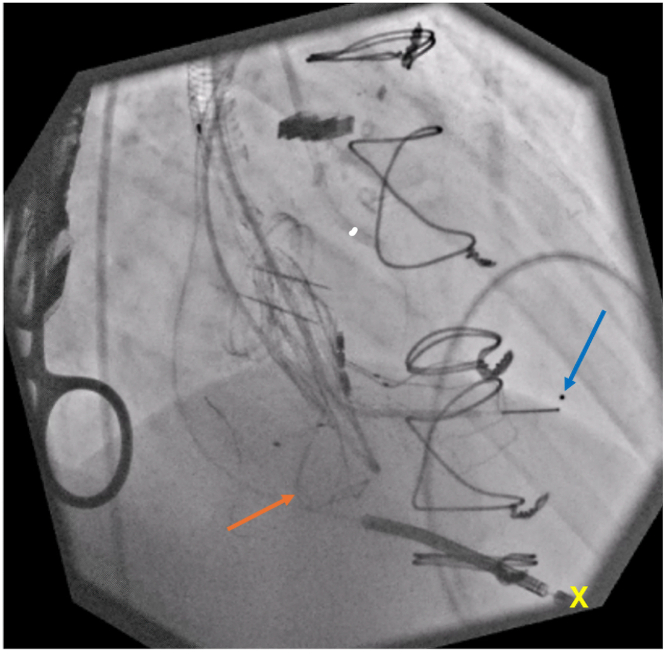
Unsheathed but Constrained L-Shaped Laplace Device Positioning Before Full Expansion Blue arrow indicates the distal tip of the Laplace delivery system, orange arrow indicates the posterior flap, and yellow X indicates the right ventricular apex.

In May 2025, the patient underwent TTVR with the Laplace TTVR system. The procedure was performed under general anesthesia with fluoroscopic and transesophageal echocardiographic guidance. Percutaneous access to the right internal jugular vein was made for introduction of the system. A 0.014-in coronary wire was advanced to the distal right coronary artery and used as a reference marker to identify the tricuspid annular plane. Due to the presence of multiple ICD leads, a steerable introducer (Agilis N x T; Abbott Cardiovascular) was used to help position a 0.035-in guidewire into the pulmonary artery. The Laplace TTVR delivery system was then introduced over the guidewire and advanced into the right atrium. Unsheathing was then performed, after which the constrained valve was exposed in the right atrium. The unsheathed device was then advanced under fluoroscopic guidance until the posterior flap and engagers were below the annulus (Figure 4), with this positioning confirmed via transesophageal echocardiography.

**Figure 5:**
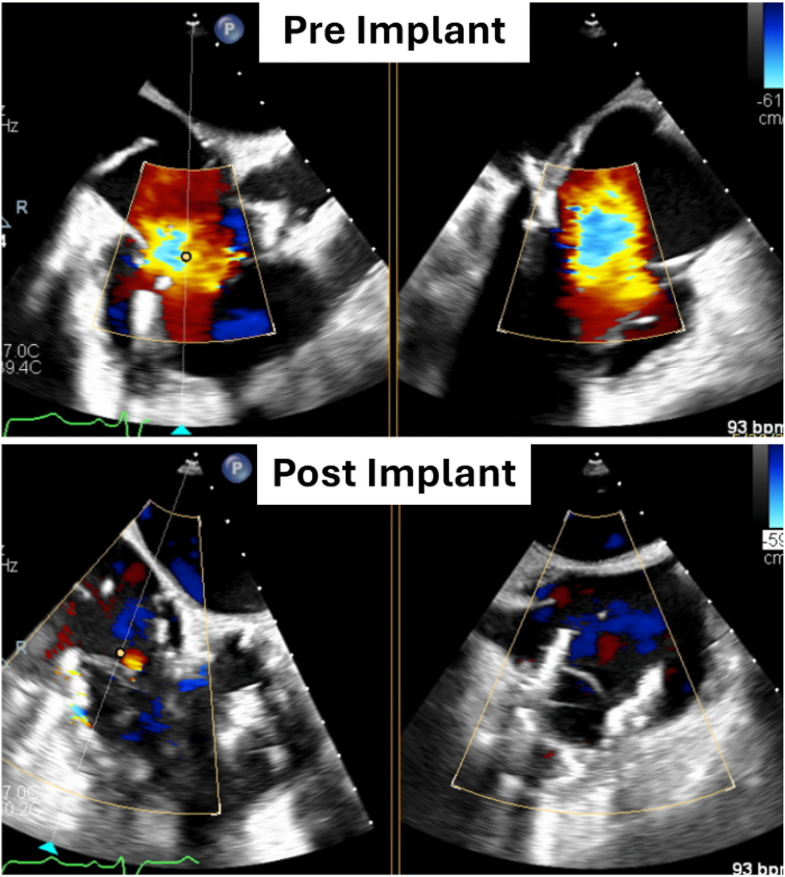
Transesophageal Echocardiography Before (Torrential Tricuspid Regurgitation) and After (None/Trace Tricuspid Regurgitation) Transcatheter Tricuspid Valve Replacement

The Laplace constrained valve was then fully expanded to assess full functionality of the prosthesis. Due to insufficient valve expansion noted on fluoroscopy, the valve was recollapsed, repositioned, and redeployed. Transesophageal echocardiography confirmed proper position and function of the valve with no evidence of any paravalvular leak (Figure 5). The valve was then released from the delivery catheter system. The right internal jugular access site was successfully closed using a double Perclose technique.^1^

## Outcome and Follow-Up

TTE performed before discharge demonstrated a well-positioned tricuspid bioprosthesis with normal function and no paravalvular leak. The postoperative course was uneventful, and the patient was discharged on postprocedural day 2. At 1-month follow-up, TTE showed similar findings. Six-minute walk distance improved to 449 m from a baseline 6-minute walk distance of 302 m. Kansas City Cardiomyopathy Questionnaire assessment improved from 42.2 to 81.8 points, consistent with a meaningful improvement in overall health status and quality of life. At 3- and 6-month follow-up, all the improvements observed at 1 month were sustained (Table 1).

**Table 1:** Baseline, 1-Month, 3-Month, and 6-Month Follow-Up Data Summary

	Baseline	1 mo	3 mo	6 mo
TR (TTE)	Torrential	None/trace	None/trace	None
NYHA functional class	II	II	II	I
KCCQ	42.2	81.8	77.1	93.3
6-min walk distance, m	302	449	477	470

KCCQ = Kansas City Cardiomyopathy Questionnaire; TR = tricuspid regurgitation; TTE = transthoracic echocardiography.

## Discussion

Transcatheter approaches for treatment of severe TR include T-TEER, TTVR, and CAVI. T-TEER is considered in patients with smaller coaptation gaps (ideally <7-10 mm), minimal leaflet tethering, or in those who cannot tolerate anticoagulation, which is required for TTVR. CAVI is usually reserved for patients with tricuspid valve anatomy that is unsuitable for TTVR or T-TEER. Although it reduces the symptoms of systemic venous congestion, it does not modify the degree of TR. TTVR promises complete elimination of TR but carries a higher procedural risk profile, requires long-term anticoagulation, and can acutely worsen RV function when RV to pulmonary arterial coupling is poor.^2^ Cardiac implantable electronic device (CIED) leads are common in patients with severe TR and present additional challenges for both T-TEER and TTVR, including risk of entanglement or lead fracture, respectively. In this young patient with torrential TR and normal baseline RV systolic function, TTVR was favored.

The EVOQUE system is the only TTVR device approved by the Food and Drug Administration, and it is best suited for patients with an RA height (distance from tricuspid valve annular plane to the RA roof) of at least 55 to 60 mm to ensure coaxial delivery when inserted via a standard right transfemoral approach.^3^ Our patient's RA height was only 39.4 mm^2^. Small RA dimensions limit the maneuverability of the delivery system, potentially precluding optimal delivery trajectory, interfering with coaxial alignment, and increasing the risk of deployment deep into the RV cavity.^3^^,^^4^ Although a left transfemoral or transjugular approach may allow for appropriate placement in patients with smaller RA height due to a more favorable angle of approach, a transjugular approach was unsuccessful in our patient.

The investigational TTVR system used in this case features an L-shaped configuration that is designed to shorten the working length of the delivery system after unsheathing in the right atrium (Figure 4). Coaxial alignment with respect to the tricuspid valve annulus is not required during tracking or deployment, and the device can be recaptured, repositioned, or even removed after deployment. These features allowed for safe and successful deployment of the TTVR in this patient with unique anatomical constraints. Although there was no evidence of deleterious interaction of the TTVR device with the patient's CIED leads during or after the procedure, the long-term effects of lead entrapment and feasibility of extraction are unknown.

This case underscores some of the common anatomical and technical challenges encountered in patients undergoing TTVR that may limit the use of currently available devices. The Laplace TTVR, and other systems under development, may expand the number of patients eligible for TTVR by addressing some of these challenges. Devices that successfully reduce the risk of deleterious interaction with CIED leads will also be important, because these are commonly encountered in patients with severe TR. Although our patient had an excellent outcome, this report describes a single case using an investigational device in a center with expert operators. Further experience is required to define procedural safety, device durability, and comparative effectiveness across a broader subset of patients.

## Conclusions

This case demonstrates the feasibility of TTVR in a patient with torrential TR and limited RA working room using the investigational Laplace device. Implantation resulted in resolution of TR without complications and sustained improvements in functional status and symptoms at 6-month follow-up. Ongoing development of novel devices promises to expand the number of patients eligible for TTVR; however, attention to procedural safety and long-term durability are essential.

## Funding Support and Author Disclosures

Drs Mishell and Rassi have received institutional research funding from Edwards Lifesciences and Laplace Interventional. Dr Sannino has received grant support from Cardiovalve, Edwards Lifesciences, and Medtronic. All other authors have reported that they have no relationships relevant to the contents of this paper to disclose.
